# Supplementary material for: Direct Detection of Inhomogeneity in CVD-Grown 2D TMD Materials via K-Means Clustering Raman Analysis
Source: Nanomaterials (Basel). 2022 Jan 27;12(3):414. doi: 10.3390/nano12030414 (PMC8840665; doi:10.3390/nano12030414)
Supplement: Supplementary file 1 [file nanomaterials-12-00414-s001.zip › nanomaterials-1481667-supplementary.pdf]

Supporting Information

Direct Detection of Inhomogeneity in CVD-Grown 2D TMD Materials via K-Means Clustering Raman Analysis

Hang Xin [1,2,3] , Jingyun Zhang [1,2,3]*, Cuihong Yang [1,2,3] and Yunyun Chen [1,2,3]

1 School of Physics & Optoelectronic Engineering, Nanjing University of Information Science & Technology, Nanjing 210044, China; 20191217010@nuist.edu.cn (H.X.); yangcuihong1978@163.com (C.Y.); yunqq321@sina.cn (Y.C.)
2 Jiangsu Key Laboratory for Optoelectronic Detection of Atmosphere and Ocean, Nanjing University of Information Science & Technology, Nanjing 210044, China
3 Jiangsu International Joint Laboratory on Meterological Photonics and Optoelectronic Detection, Nanjing University of Information Science & Technology, Nanjing 210044, China
* Correspondence: zhangjingyun@nuist.edu.cn

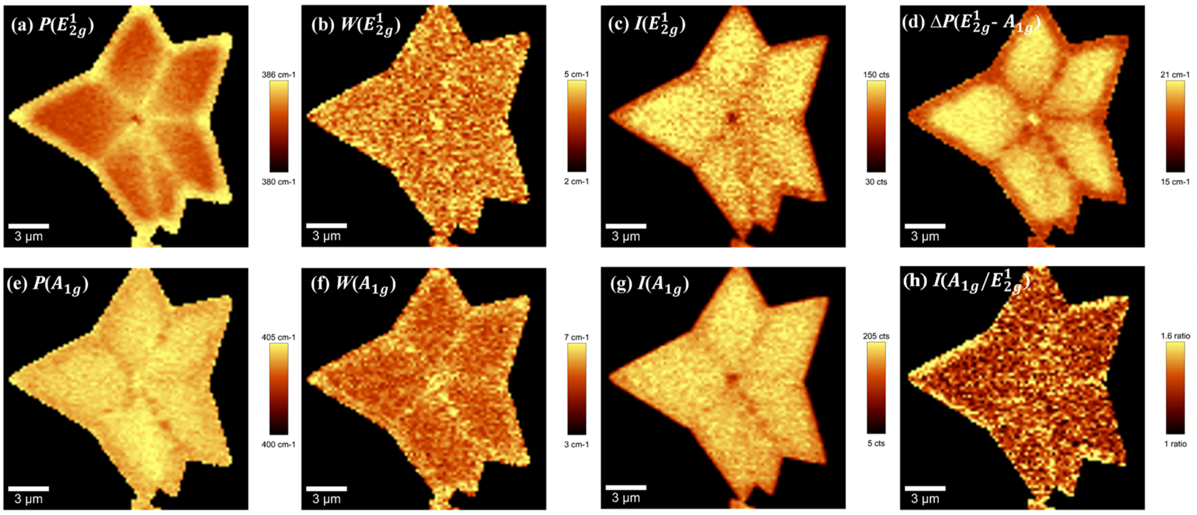

Figure S1. (**a**-**c**) and (**e**-**g**) are Raman images of monolayer polycrystalline MoS_2 plotted as a function of the peak position (P), width (W), and intensity (I) of E_{2g}^1 and A_{1g} modes. The difference between E_{2g}^1 and A_{1g} modes in peak position and relative peak intensities are calculated and plotted in (d) and (**h**).

Cluster Name	$P(E_{2g}^1)$ (cm^{-1})	$W(E_{2g}^1)$ (cm^{-1})	$I(E_{2g}^1)$ (a.u.)	$P(A_{1g})$ (cm^{-1})	$W(A_{1g})$ (cm^{-1})	$I(A_{1g})$ (a.u.)	$\Delta P(A_{1g}\text{-}E_{2g}^1)$ (cm^{-1})	$I\,(A_{1g}/E_{2g}^1)$
Cluster 1	**387.96**	4.55	108.50	406.99	**6.26**	154.86	19.03	1.43
Cluster 2	**387.29**	4.39	127.91	407.25	**5.92**	177.87	19.96	1.39
Cluster 3	**386.63**	4.34	140.65	407.25	**5.70**	185.79	20.61	1.32

Table S1. Raman parameters of the cluster 1-3 in monolayer polycrystalline MoS_2 sample fitted with Lorentz function.

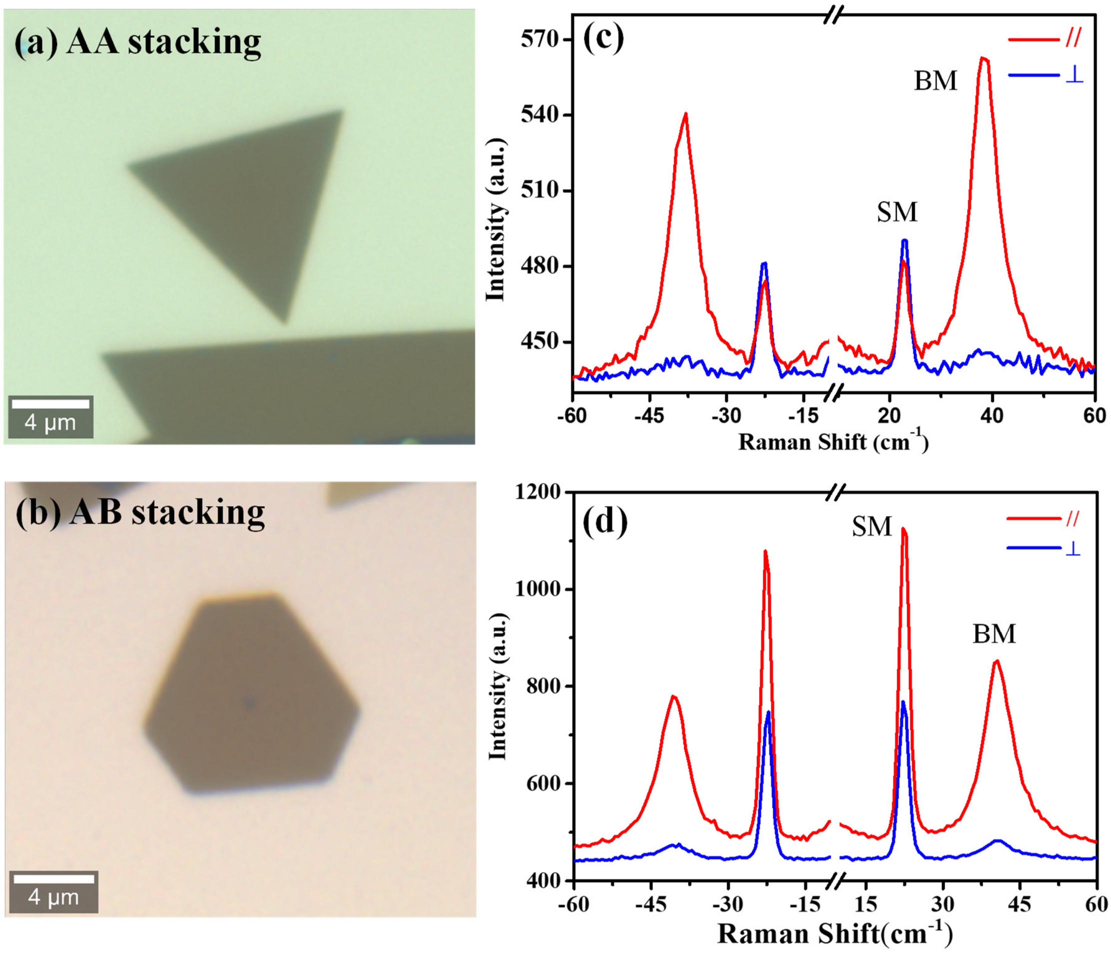

Figure S2. Stacking identification of AA and AB bilayer MoS_2 by polarized Raman spectroscopy. (**a**-**b**) Optical image of CVD-grown bilayer MoS_2 and (**c**-**d**) the correlated Stoke and anti-Stoke low wavenumber Raman spectra measured under parallel (//) and cross (⊥) polarization configurations, respectively. The AA and AB stacking orders could be extracted from the relative intensities between the shearing mode (SM) and breathing mode (BM) at 22.5 and 40.5 cm^{-1} in excellent agreement with X. Yan *et al*'s work.[1]

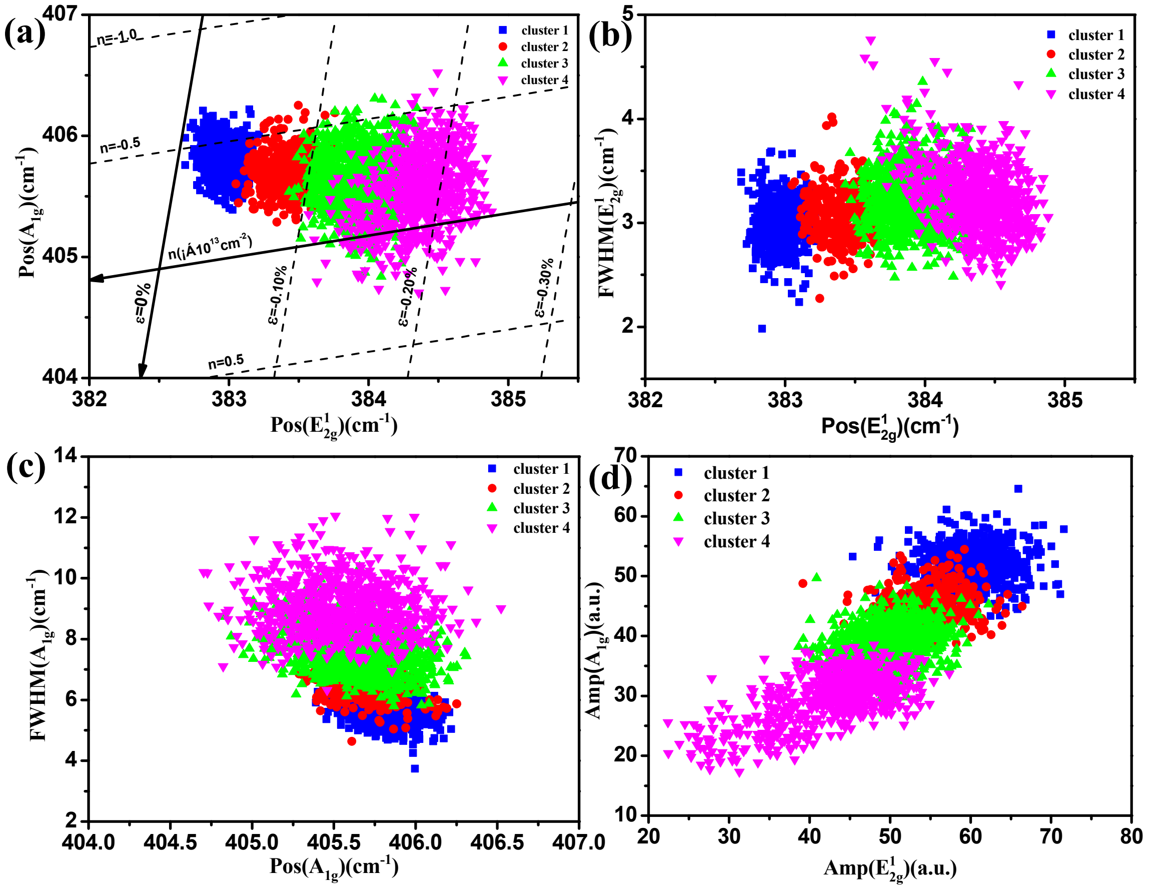

Figure S3. Correlative analysis of Raman spectral features to quantitatively evaluate the contribution of strain and doping effects on bilayer MoS_2 with AA-stacking. (**a**) The 2D correlative analysis of Pos(E_{2g}^1) and Pos(A_{1g}) in cluster 1-4. (**b**) Correlation plot of FWHM(E_{2g}^1) *vs* Pos(E_{2g}^1). (**c**) Correlation plot of FWHM(A_{1g}) *vs* Pos(A_{1g}). (**d**) Correlation plot of AMP(A_{1g}) *vs* AMP(E_{2g}^1).

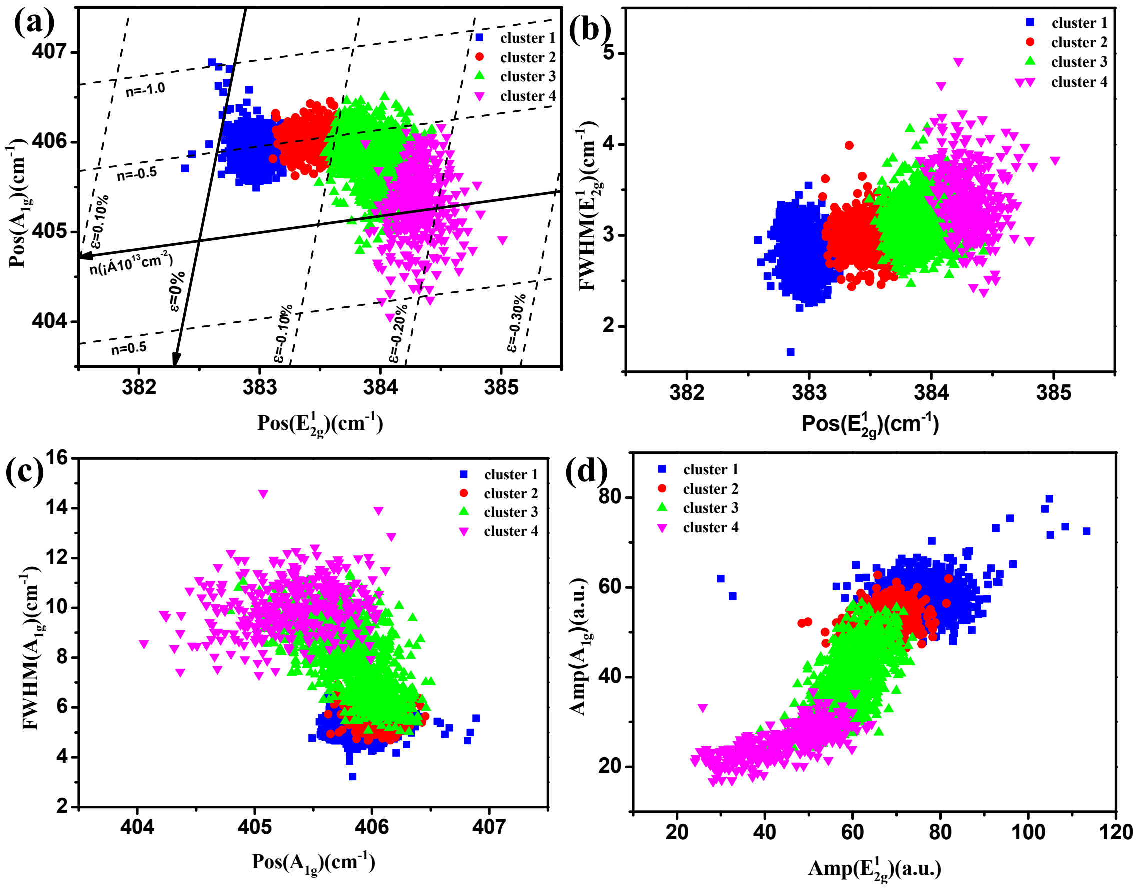

Figure S4. Correlative analysis of Raman spectral features to quantitatively evaluate the contribution of strain and doping effects on monolayer MoS_2 with AB stacking. The 2D correlative analysis of (**a**) Correlation plots of Pos(E^1_{2g}) and Pos(A_{1g} in cluster 1-4. (**b**)~(**d**) Correlation plots of FWHM(E^1_{2g}) *vs* Pos(E^1_{2g}), FWHM(A_{1g}) *vs* Pos(A_{1g}) and AMP(A_{1g}) *vs* AMP(E^1_{2g}), respectively.

1 Yan, J. *et al.* Stacking-Dependent Interlayer Coupling in Trilayer MoS(2) with Broken Inversion Symmetry. *Nano Lett* **15**, 8155-8161, doi:10.1021/acs.nanolett.5b03597 (2015).
